# Acute liver failure and seizures as a consequence of regorafenib exposure in advanced rectal cancer

**DOI:** 10.1186/s13104-015-1502-4

**Published:** 2015-10-05

**Authors:** Soundouss Raissouni, Zarqa Quraishi, Mohammed Al-ghamdi, Jose Monzon, Patricia Tang, Michael M. Vickers

**Affiliations:** Tom Baker Cancer Centre, 1331, 29th Street, NW, Calgary, AB T2N 4N2 Canada; King Saud University, Riyadh, Saudi Arabia

**Keywords:** Regorafenib, Seizures, Colorectal cancer, Liver failure

## Abstract

**Background:**

Regorafenib is a multi-targeted tyrosine kinase inhibitor approved for use in refractory colorectal cancer. We report the first case of seizures secondary to acute liver failure, shortly after initiation of regorafenib in a patient with advanced rectal carcinoma.

**Case presentation:**

A 64 year-old Caucasian female presented with confusion and generalized tonic–clonic seizures, 5 days after initiation of regorafenib for advanced rectal cancer. Investigations revealed significant elevations in bilirubin and alanine aminotransferase. No other cause for seizures and liver dysfunction were found. After interruption of regorafenib, no further seizures occurred, the symptoms of confusion resolved and liver function returned to normal.

**Conclusions:**

We report the first case of regorafenib-induced acute liver failure resulting in seizures. We suggest early monitoring for side effects, both clinically and biochemically after initiation of regorafenib.

## Background

Regorafenib is a novel oral multi-kinase inhibitor that targets oncogenesis, tumor angiogenesis and the tumor microenvironment [1]. In cellular kinase phosphorylation assays, regorafenib inhibited VEGFR-1, -2 and -3, FGFR1, PDGFR-b, KIT, RET, RAF-1, BRAF and BRAFV600E [2, 3]. Currently, regorafenib is indicated as monotherapy for the treatment of refractory advanced colorectal cancer (ACRC), based on a large phase III randomised clinical trial (CORRECT) [4] as well as in gastrointestinal stromal tumors (GIST) after failure of imatinib and sunitinib [5]. The most common treatment-related adverse events (any grade) with regorafenib reported in the CORRECT trial included hand-foot skin reaction (47 %), fatigue (47 %), diarrhea (34 %), anorexia (30 %), voice changes (29 %), hypertension (28 %) mucocitis (27 %), and rash (26 %). Liver function abnormalities were also more common in patients receiving regorafenib and included elevations in alanine aminotransferase (ALT), aspartate aminotransferase (AST) and bilirubin [4]. One case of fatal liver dysfunction was also observed. Conversely, central nervous system (CNS) toxicity has rarely been reported with this agent [3, 6]. We report a case of acute liver failure complicated by seizure shortly after initiation of regorafenib in a patient with advanced rectal carcinoma.

## Case presentation

**Fig. 1:**
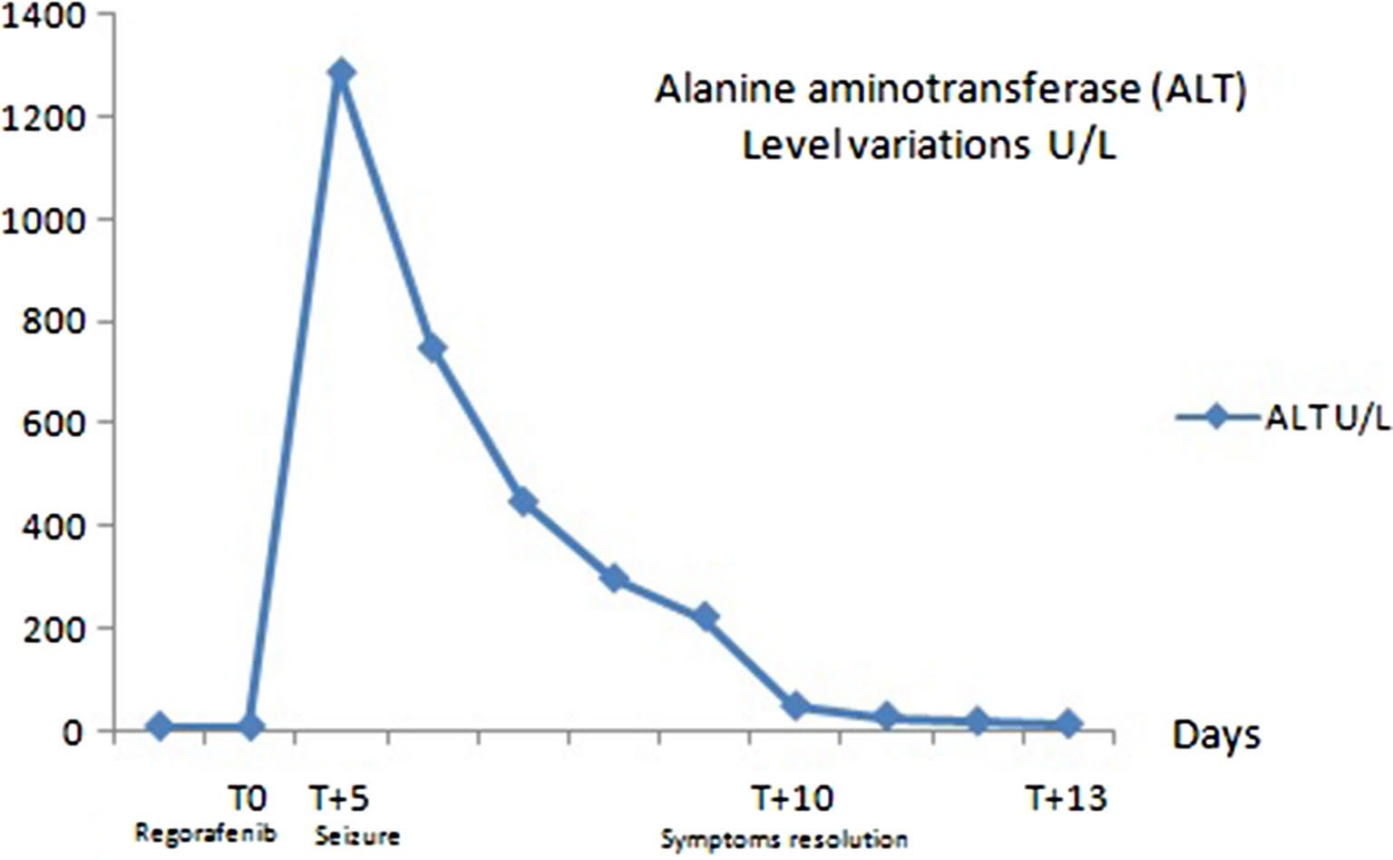
Alanine aminotransferase (ALT) level variations (U/L)

**Fig. 2:**
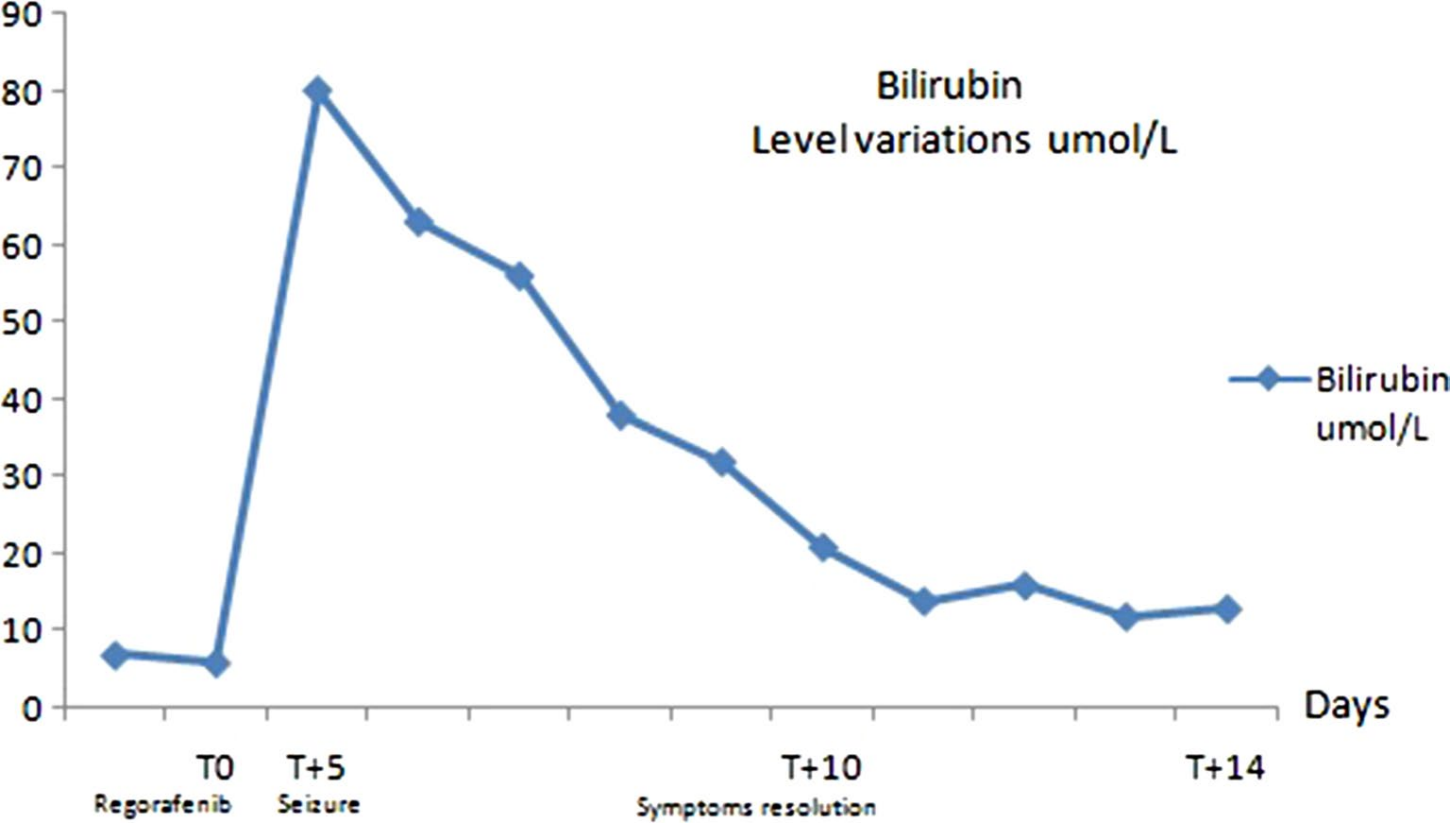
Bilirubin level variations (umol/L)

A 64 year-old Caucasian female was followed at our institution since June 2009 for rectal cancer. Her past medical history was remarkable for acute kidney injury of unknown etiology in 2009 (resolved), prior smoking history and moderate alcohol intake for 35 years (discontinued in 2009). Initial staging of her rectal cancer revealed a cT4N2M0, and she received neoadjuvant chemoradiation (45 Gy concurrent with continuous infusional fluorouracil at a dose of 225 mg/m^2^ per day) from July–August 2010. Resection of the primary tumor was attempted in November 2010, but was unsuccessful due to progressive local disease and a diverting colostomy was placed. A Positron emission tomography/computed tomography (PET/CT) scan in September 2010 revealed pulmonary and liver metastasis. Subsequent lines of chemotherapy included FOLFIRI (folinic acid, fluorouracil and irinotecan) plus bevacizumab with initially stable disease. At progression, she received FOLFOX (folinic acid, fluorouracil and oxaliplatin) for 4 months followed by 15 cycles of Panitumumab (KRAS wild type tumor) with initially stable disease. Throughout her therapies there was no evidence of CNS toxicity or liver toxicity and the patient maintained an ECOG performance status of 0. In January 2014, re-staging scans showed progressive local and distant disease. The patient initiated regorafenib at the dose of 120 mg daily (25 % dose reduction as patient concerned about potential toxicities). Five days after starting regorafenib she presented to the emergency department with diarrhea, three episodes of generalized tonic–clonic seizures and decreased level of consciousness. On admission the patient was afebrile, mildly jaundiced, had a blood pressure of 148/90 mmHg, heart rate of 90 beats per minute, oxygen saturation of 97 % on room air, and found to have grade 3 encephalopathy, and a Glasgow coma score of 10. No other focal neurologic deficits were identified. Admission blood work revealed normal electrolytes aside from mild hypokalemia of 3.1 mEq/L and normal renal function. There was evidence of grade 3 (Common Terminology Criteria for Adverse Events (CTCAEv3.0)) hyperbilirubinemia (80 umol/L), grade 4 elevation of ALT (1290 U/L), grade 1 elevation in alkaline phosphatase (267 U/L) and grade 3 International normalized ratio (INR) elevation at 2.6. Complete blood count was unremarkable except for a leukocytosis of 22 × 10^9^/L. Blood cultures and urine cultures were negative and initial enhanced computed tomography of the head was unremarkable. Initial management, before obtaining the results of the initial workup, included discontinuation of regorafenib, administration of benzodiazepines, a loading dose of phenytoin, and treatment with acyclovir and broad spectrum antibiotics. Cytology from a lumbar puncture was negative (cytology negative and negative for infectious encephalitis) and magnetic resonance imaging (MRI) of the brain revealed small areas of increased T2 signal seen in the hemispheric sulci in the region of the occipital poles, right frontal convexity, and right parietal parafalcine location, but did not reveal the classic changes of reversible posterior leukoencephalopathy syndrome (RPLS). Electroencephalography (EEG) revealed runs of frontal intermittent rhythmic delta activity and rare triphasic waves. In addition, there was generalized slowing consistent with diffuse abnormal brain function suggestive of a moderate encephalopathy related to a metabolic etiology. A CT scan of the abdomen did not show any sign of biliary obstruction. Despite only having hypokalemia at presentation, she required daily electrolyte replacement to correct grade 3 hypomagnesemia, grade 2 hypocalcemia, grade 4 hypokalemia and grade 3 hypophosphatemia. As a consequence of electrolytes abnormalities, electrocardiogram revealed prolonged QTc at 519 ms, T wave inversion in inferior and lateral leads. Liver function at discharge (day 10) improved significantly with normalization of bilirubin (14 umol/L) and mild elevations in ALT (54 U/L) and INR (1.2) (Figs. 1, 2). Seventeen days after admission, ALT (37 U/L) and bilirubin (16 umol/L) returned to normal. Confusion and memory loss resolved and there were no further seizures after the day of admission.

## Discussion

Acute liver failure (ALF) is a syndrome involving sudden and severe loss of hepatocyte function due to hepatocyte necrosis [7]. This diagnosis is characterized by acute liver dysfunction, hepatic encephalopathy and coagulopathy. Patients with rapid onset of jaundice are at the highest risk of cerebral complications, such as asterixis, delirium, seizures and coma. The exact pathophysiology of these cerebral effects is yet to be determined, however may involve cerebral edema/astrocyte swelling, ammonia accumulation, oxidative stress and blood–brain barrier dysfunction [8]

Our case is consistent with regorafenib exposure leading to acute liver failure and subsequent hepatic encephalopathy and seizures. Prior to initiation of regorafenib, the patient’s liver function was normal, but acutely deteriorated with significant elevations in ALT, bilirubin and the development of hepatic encephalopathy and coagulopathy. In addition, the MRI and EEG findings were not consistent RPLS which was considered in this case and has been reported with other anti-angiogenic agents [9]. Recently, Zaw et al. [10] reported a case of grand mal seizure shortly after initiation of regorafenib in a patient treated with metastatic colon carcinoma. Investigations in that case confirmed the diagnosis of RPLS with characteristic MRI findings. In addition, this patient presented with elevated blood pressure (>200/100 mmHg) in the absence of liver dysfunction [10]. In contrast, our patient was not overly hypertensive on admission, did not have visual defects nor was the MRI suggestive of RPLS. One seizure was also reported in the CORRECT trial although the details of this case are unknown [4]. To our knowledge, our case is the first to describe regorafenib-induced seizure as a result of acute liver failure.

In the CORRECT trial, liver function test abnormalities were common and included elevations in ALT (any grade: 45.2 %; grade 3/4: 5.5 %), AST (any grade: 65 %; grade: 3/4 5.9 %) and bilirubin (any grade: 44.6 %; grade: 3/4 12.2 %) (see Web appendix, Online Table 6) [4]. One patient with liver metastases had progressive liver dysfunction 43 days after initiation of regorafenib with a fatal outcome. In both the CORRECT and GRID trials liver function was monitored every 2 weeks, with closer monitoring of AST, ALT and bilirubin (twice weekly for 2 weeks and then weekly for at least 4 weeks) after the occurrence of liver function abnormalities. Despite an initial dose reduction, our patient experienced acute liver failure 5 days after starting regorafenib. Toxicity of this degree would not be captured with the current recommendations of every two weekly blood monitoring and as such, we suggest close monitoring of patients after initiation of regorafenib with early clinical and laboratory assessment within 3–5 days.

## Conclusion

In conclusion, we report a case of regorafenib-induced acute liver failure precipitating seizures in advanced rectal cancer. Since regorafenib is indicated in the refractory setting of colorectal cancer treatment and has shown a modest survival benefit, consideration of patients’ quality of life and avoidance of significant side effects must be paramount. Close monitoring for side effects, both clinically and biochemically is indicated.

### Consent

Written informed consent was obtained from the patient for publication of this Case Report and any accompanying images. A copy of the written consent is available for review by the Editor-in-Chief of this journal

## Abbreviations

ACRC: advanced colorectal cancer
GIST: gastrointestinal stromal tumors
ALT: alanine aminotransferase
AST: aspartate aminotransferase
INR: international normalized ratio
CNS: central nervous system
PET/CT: positron emission tomography/computed tomography
MRI: magnetic resonance imaging
RPLS: reversible posterior leukoencephalopathy syndrome
EEG: electroencephalography
ECG: electrocardiogram
CTCAE: common terminology criteria for adverse events
